# Essentials of Physics

**Published:** 1892-06

**Authors:** 


Essentials of Physics, arranged in the form of questions and answers. Prepared especially for Students of Medicine. By Fred J. Brockway, M. D., Assistant Demonstrator of Anatomy at the College of Physicians and Surgeons, New York. With 155 illustrations. Philadelphia: W. B. Saunders, 913 Walnut street. 1892.

This is one of the most valuable books in the Saunders' Question Series. There are many reasons why medical students cannot devote as much time to the study of physics as they should, hence, it is well to have this subject condensed as much as possible. Dr. Brockway has accomplished this feat in the book before us without doing violence to its importance or otherwise belittling the subject. We advise every medical student who feels himself deficient in the study of physics to possess this valuable volume.

